# Novel insight into the lipid network of plasma extracellular vesicles reveal sex-based differences in the lipidomic profile of alcohol use disorder patients

**DOI:** 10.1186/s13293-024-00584-5

**Published:** 2024-01-25

**Authors:** Carla Perpiñá-Clérigues, Susana Mellado, Cristina Galiana-Roselló, María Fernández-Regueras, Miguel Marcos, Francisco García-García, María Pascual

**Affiliations:** 1 Computational Biomedicine Laboratory, Príncipe Felipe Research Center, C/Eduardo Primo Yúfera, 3, 46012 Valencia, Spain; 2 Department of Physiology, School of Medicine and Dentistry, University of Valencia, Avda. Blasco Ibáñez, 15, 46010 Valencia, Spain; 3 Department of Inorganic Chemistry, Institute of Molecular Science, University of Valencia, 46980 Paterna, Spain; 4 Hospital Universitario de Burgos, 09006 Burgos, Spain; 5 Hospital Universitario de Salamanca, 37007 Salamanca, Spain; 6 Department of Internal Medicine, University Hospital of Salamanca, University of Salamanca, Institute of Biomedical Research of Salamanca (IBSAL), 37007 Salamanca, Spain

**Keywords:** Lipidomics, Lipid network, Extracellular vesicles, Alcohol use disorder, Sex-based differences

## Abstract

**Background:**

Alcohol use disorder (AUD) is one of the most common psychiatric disorders, with the consumption of alcohol considered a leading cause of preventable deaths worldwide. Lipids play a crucial functional role in cell membranes; however, we know little about the role of lipids in extracellular vesicles (EVs) as regulatory molecules and disease biomarkers.

**Methods:**

We employed a sensitive lipidomic strategy to characterize lipid species from the plasma EVs of AUD patients to evaluate functional roles and enzymatic activity networks to improve the knowledge of lipid metabolism after alcohol consumption. We analyzed plasma EV lipids from AUD females and males and healthy individuals to highlight lipids with differential abundance and biologically interpreted lipidomics data using LINEX^2^, which evaluates enzymatic dysregulation using an enrichment algorithm.

**Results:**

Our results show, for the first time, that AUD females exhibited more significant substrate-product changes in lysophosphatidylcholine/phosphatidylcholine lipids and phospholipase/acyltransferase activity, which are potentially linked to cancer progression and neuroinflammation. Conversely, AUD males suffer from dysregulated ceramide and sphingomyelin lipids involving sphingomyelinase, sphingomyelin phosphodiesterase, and sphingomyelin synthase activity, which relates to hepatotoxicity. Notably, the analysis of plasma EVs from AUD females and males demonstrates enrichment of lipid ontology terms associated with “negative intrinsic curvature” and “positive intrinsic curvature”, respectively.

**Conclusions:**

Our methodological developments support an improved understanding of lipid metabolism and regulatory mechanisms, which contribute to the identification of novel lipid targets and the discovery of sex-specific clinical biomarkers in AUD.

**Supplementary Information:**

The online version contains supplementary material available at 10.1186/s13293-024-00584-5.

## Plain Language Summary

Alcohol use disorder (AUD) is one of the most common psychiatric disorders, with the consumption of alcohol considered a leading cause of preventable deaths worldwide. Lipids play a crucial functional role in cell membranes; however, we know little about the role of lipids in extracellular vesicles (EVs) as regulatory molecules and disease biomarkers. We employed a sensitive lipidomic strategy to characterize lipid species from the plasma EVs of AUD patients to evaluate functional roles and enzymatic activity networks to improve the knowledge of lipid metabolism after alcohol consumption. We analyzed plasma EV lipids from AUD females and males and healthy individuals to highlight lipids with differential abundance and biologically interpreted lipidomics data using LINEX^2^, which evaluates enzymatic dysregulation using an enrichment algorithm. Our results show, for the first time, that AUD females exhibited more significant substrate-product changes in lysophosphatidylcholine/phosphatidylcholine lipids and phospholipase/acyltransferase activity, which are potentially linked to cancer progression and neuroinflammation. Conversely, AUD males suffer from dysregulated ceramide and sphingomyelin lipids involving sphingomyelinase, sphingomyelin phosphodiesterase, and sphingomyelin synthase activity, which relates to hepatotoxicity. Notably, the analysis of plasma EVs from AUD females and males demonstrates enrichment of lipid ontology terms associated with “negative intrinsic curvature” and “positive intrinsic curvature”, respectively. Our methodological developments support an improved understanding of lipid metabolism and regulatory mechanisms, which contribute to the identification of novel lipid targets and the discovery of sex-specific clinical biomarkers in AUD.

**Supplementary Information:**

The online version contains supplementary material available at 10.1186/s13293-024-00584-5.

## Highlights

- AUD induced sex-based differences in lipid profiles related to EVs biogenesis and may underlie inflammatory and neurodegenerative responses.
- Females with AUD display significant alterations in lysophosphatidylcholine/phosphatidylcholine lipids and phospholipase/acyltransferase activity, suggesting associations with cancer progression and neuroinflammation.
- Males with AUD display significant alterations in sphingomyelinase, sphingomyelin phosphodiesterase, and sphingomyelin synthase activity, which relates to hepatotoxicity.
- The properties of the lipidome, as determined by the LION algorithm, indicate sex-based differences in the modifications of lipids associated with membrane remodeling and lipid-mediated signaling in EVs from AUD patients.
- The study employs an innovative approach, utilizing bioinformatic analysis of lipidomic data, to identify novel lipid targets and uncover sex-specific clinical biomarkers in AUD.

**Supplementary Information:**

The online version contains supplementary material available at 10.1186/s13293-024-00584-5.

## Background

Alcohol use disorder (AUD) is a chronic disease characterized by unhealthy alcohol use and several neurobiological features, such as positive reinforcement, a compulsive search for alcohol, and a negative emotional state following abstinence from alcohol use [1]. AUD, which comprises a constellation of symptoms (including withdrawal, tolerance, and craving), is a significant public health issue that has recently suffered a significant and alarming increase in prevalence. Alcohol use is estimated to cause approximately three million deaths globally each year and constitutes a significant factor for morbimortality [2]. Alcohol-induced adverse consequences to health include alcohol-associated liver disease, hepatocellular carcinoma, non-liver neoplasms, physical injury, cardiac disease, and psychiatric disorders. Alcohol misuse also significantly affects workforce productivity and elevates direct and indirect economic costs, as many of those affected by alcohol misuse are in the most productive years of their lives [2].

Importantly, the neurobiology and pathological consequences associated with AUD are strongly influenced by biological factors, mainly related to sex. Males have higher rates of physical and behavioral problems, whereas females have a higher risk of developing psychiatric and physical comorbidities [3, 4]. In the brain, evidence also supports that intracranial gray matter was smaller in alcoholic women than in men, whereas microstructural integrity of cortical and callosal white matter was disrupted to similar extents in both sexes [3]. In addition, it has been shown in recent years that alcohol affects the neuroimmune signaling and synaptic function differently in females and males. Females are more vulnerable to the neurotoxic effects of alcohol, and show more exacerbated neuroinflammatory changes than their male counterparts [5]. These findings highlight the need to elucidate the underlying sex-specific pathophysiological processes of AUD in order to develop personalized approaches for the prevention and treatment.

Extracellular vesicles (EVs) are diverse, nanoscale membrane vesicles actively released by most, if not all, cells. EVs are increasingly recognized as important mediators of intercellular communication and circulating biomarkers for disease diagnosis/prognosis [6]. A range of studies has demonstrated the role of EVs in physiological processes and pathological conditions, such as inflammation, cancer, and neurodegeneration [7, 8]. While recent research has provided examples of the roles of the DNA, RNA, and protein content of EVs in biological processes, we know relatively little regarding lipids. We recently demonstrated that binge-like ethanol drinking induces a differential enrichment of lipid species in plasma EVs isolated from human female adolescents compared to males [9]; furthermore, we found that these lipid species participate in EV formation, release, and uptake, as well as inflammatory immune responses.

Lipids represent crucial components of cell membranes and participate in a range of cellular functions. Understanding how changes to lipids caused by pathological conditions, environmental factors, or treatments impact cellular processes represents a critical challenge that will provide new insights into potential disease mechanisms [10]. Mass spectrometry-based lipidomics combined with dedicated computational tools [11] represents a powerful tool for identifying and quantifying lipids in cells, tissues, or bodily fluids [12]. Although recent reports have focused on analyzing lipid composition and abundance, the fact that thousands of lipid species interact via multiple pathways and networks remains a challenging aspect of this type of analysis. Evaluating and understanding changes in lipid networks in response to cellular environment alterations associated with disease development represents a crucial means of deciphering cell metabolism and related molecular mechanisms [13]. In this sense, new bioinformatic tools such as the lipid network explorer (LINEX^2^), which combines lipid classes and lipid metabolic reactions, can comprehensively approach the interpretation of lipidomics data [11]. These methodological developments have allowed for a better understanding of lipid metabolism and regulatory mechanisms, thereby contributing to identifying novel lipid targets and discovering clinical biomarkers [14, 15].

Taking into account the novel approach based on bioinformatic analysis and the critical roles of EV lipids as biomarkers, here, we employed a highly sensitive lipidomic-based strategy to characterize lipid species from EVs isolated from the plasma of AUD females and males and evaluate the differential functional roles and enzymatic activity networks of EV lipids to improve our understanding of how alcohol consumption impacts lipid metabolism. We demonstrate sex-based differences in EV lipid composition induced by alcohol consumption, which impacts species-/class-level abundance and lipid metabolic networks. Furthermore, we discovered that the identified lipids often had roles in EV biogenesis and/or inflammatory/neurodegenerative responses. Importantly, we have made all data and results openly available on a web-based platform (https://bioinfo.cipf.es/sal-chronics).

## Methods

### Human subjects

The eleven AUD patients (according to DSM-5 criteria) included in this study (six males and five females) were referred to the Alcoholism Unit of the University Hospital of Salamanca (Spain) [16]. The median age of AUD males and females was 47.83 and 40.00 years, respectively. All patients in this group actively drank ≥ 90 g of ethanol/day until entering the study. All patients had normal prothrombin time, hemoglobin concentration, and albumin serum levels and tested negative for hepatitis B surface antigen and hepatitis C antibodies. Patients did not suffer from other chronic/acute conditions that could alter the study results and were not polydrug abusers. Advanced liver disease was excluded based on clinical, analytical, and ultrasonographic studies: individuals displaying physical stigmata of chronic liver disease (e.g., cutaneous signs, hepatosplenomegaly, gynecomastia, testicular atrophy, and/or muscle wasting) with liver ultrasonographic findings other than steatosis or with increased liver transaminases > 2–3 times the reference limits were excluded. Twelve healthy volunteers (six males and six females) who reported drinking < 15 g of ethanol/day were also analyzed; these volunteers all displayed normal liver function and standard hematological/biochemical test outcomes (Additional file 1: Table S1). The median age of the healthy female and male patients was 45.50 and 39.50 years, respectively. Before entering the study, all individuals gave their informed consent to participate, and the study was approved by the Ethics Committee of the University Hospital of Salamanca (Spain).

Heparin-anticoagulated peripheral blood samples were obtained from AUD and healthy patients between 9:00 and 10:00 a.m. under fasting conditions. Plasma samples were snap-frozen in liquid nitrogen and stored at − 80 °C until further use. Samples were processed for biochemical tests and EV isolation.

### EV isolation from human plasma

Plasma EVs were isolated using a total exosome isolation kit (catalog number 4484450, Invitrogen, USA), following the manufacturer’s instructions. 250 μL of initial plasma was used to isolate EVs, which were collected and frozen at − 80 °C until processing.

### EV characterization by transmission electron microscopy and nanoparticle tracking analysis

**Fig. 1:**
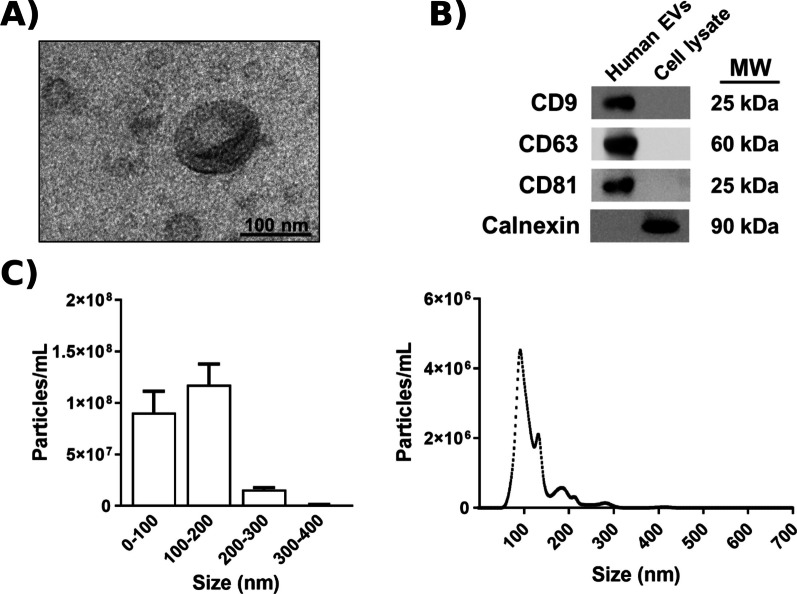
Characterization of plasma EVs. **A** Transmission electron microscopy image of human plasma EVs. **B** Analysis of the protein expression of EV markers (CD9, CD63, and CD81) in plasma EVs and astroglial cell lysates (positive control for calnexin expression). Calnexin expression was used to detect possible cytosolic protein contamination in EV samples. A representative immunoblot for each protein is shown. **C** Measurement of human EV size distribution (left) and concentration (right) by nanoparticle tracking analysis

Freshly isolated EVs were fixed with 2% paraformaldehyde and prepared for transmission electron microscopy (TEM) and nanoparticle tracking analysis (NTA) as previously described [17]. Briefly, EV preparations were examined under a FEI Tecnai G2 Spirit TEM (FEI Europe, Eindhoven, The Netherlands) with a Morada digital camera (Olympus Soft Image Solutions GmbH, Münster, Germany). The absolute size range and concentration of EVs were analyzed by NTA using a NanoSight NS300 Malvern (NanoSight Ltd., Minton Park, UK). Figures 1A and C show data regarding EV characterization by TEM and NTA.

### Western blot analysis

The Western blotting was performed to characterize plasma EVs (Fig. 1B), as previously described [18]. The primary antibodies used were anti-CD9, CD63, CD81, and calnexin antibodies (Santa Cruz Biotechnology, USA). Membranes were washed, incubated with the corresponding HRP-conjugated secondary antibodies, and developed using the ECL system (ECL Plus; Thermo Fisher Scientific). Additional file 1: Figure S1 includes a representative whole membrane for the expression of each protein.

### Lipid extraction

Lipids were extracted from equal amounts of plasma EVs (0.2 mL/sample) using a modified Folch extraction procedure. The last phase containing the lipids was transferred to fresh tubes, dry vacuumed with nitrogen, and lipids were stored at − 80 °C until further analysis. Dried samples were resuspended with isopropanol for liquid chromatography with tandem mass spectrometry (LC–MS/MS) acquisition using positive and negative ion modes.

### LC–MS/MS analysis

In fully automated quadrupole time of flight mass spectrometer (QTOF MS) acquisition mode, a pooled lipid extract representing the thirty-six samples (four conditions × nine replicates) was acquired by iterative tandem mass spectrometry (MS/MS). Detailed experimental methods for liquid chromatography (LC) and auto MS/MS were followed as previously described [19, 20] with minor modifications. Briefly, sample separation was performed using an Agilent 1290 Infinity LC system coupled to the 6550 Accurate-Mass QTOF (Agilent Technologies, Santa Clara, CA, USA) with electrospray interface (Jet Stream Technology) operating in positive-ion mode (3500 V) or negative-ion mode (3000 V) and high sensitivity mode. The optimal conditions for the electrospray interface were a gas temperature of 200 °C, drying gas flow of 12 L/min, nebulizer of 50 psi, sheath gas temperature of 300 °C, and sheath gas flow of 12 L/min. Lipids were separated on an Infinity Lab Poroshell 120 EC-C18 column (3.0 × 100 mm, 2.7 μm) (Agilent, Santa Clara, CA, USA). Under optimized conditions, the mobile phase consisted of solvent A (10 mM ammonium acetate, 0.2 mM ammonium fluoride in 9:1 water/methanol) and solvent B (10 mM ammonium acetate, 0.2 mM ammonium fluoride in 2:3:5 acetonitrile/methanol/isopropanol) using the following gradient: 0 min 70% B, 1 min 70% B, 3.5 min 86% B, 10 min 86% B, 11 min 100% B, 17 min 100% B operating at 50 °C and a constant flow rate of 0.6 mL/min. The injection volume was 5 µL for positive and negative modes.

The Agilent Mass Hunter Workstation Software was employed for data acquisition. LC/MS Data Acquisition B.10.1 (Build 10.1.48) was operated in auto MS/MS mode, and the three most intense ions (charge states, 1–2) within 300–1700 m/z mass range (over a threshold of 5000 counts and 0.001%) were selected for analysis. The quadrupole was set to a “narrow” resolution (1.3 m/z), and MS/MS spectra (50–1700 m/z) were acquired until 25,000 total counts or an accumulation time limit of 333 ms. To assure the desired mass accuracy of recorded ions, continuous internal calibration was performed during analyses using signals m/z 121.050873 and m/z 922.009798 for positive mode and signals m/z 119.03632 and m/z 980.016375 for negative mode. Additionally, all ions MS/MS [21] data were acquired on individual samples with an MS acquisition rate of three spectra/second and four scan segments (0, 10, 20, and 40 eV).

### Lipid annotator database

Five sets of five iterative MS/MS data files from pooled human cell extracts were analyzed with Lipid Annotator software 1 as the first step in the lipidomics workflow. This study used a novel software tool (Lipid Annotator) [22] with a combination of Bayesian scoring, a probability density algorithm, and non-negative least-squares fit to search a theoretical lipid library (modified LipidBlast) developed by Kind et al. [23, 24] to annotate MS/MS spectra.

Agilent MassHunter Lipid Annotator Version 1.0 was used for all other data analyses. Default method parameters were used, except only [M+H]+ and [M+NH4]+ precursors were considered for positive ion mode analysis, and only [M−H]− and [M+HAc−H]− precursors were considered for negative ion mode analysis. The Agilent MassHunter Personal Compound Database and Library (PCDL) Manager Version B.08 SP1 was used to manage and edit the exported annotations.

### Lipid identification

The lipid PCDL databases created were used for batch-targeted feature extraction using the Agilent Mass Hunter Qualitative version 10.0 on the respective batches of 36 all ions MS/MS data files. The provided “Profinder—Lipids.m” method was adapted in Mass Hunter Qualitative software with modifications previously described by Sartain et al. [20]. Data were analyzed using the Find by Formula (FbF) algorithm in MassHunter Qualitative Analysis. This approach uses a modified version of the FbF algorithm, which supports all ions MS/MS. Mass peaks in the low energy channel are first compared against the PCDL created for compounds with the same m/z values, and then a set of putative identifications is automatically compiled. For this list, the fragment ions in the MS/MS spectra from the PCDL are compared to the ions detected in the high-energy channel to confirm the presence of the correct fragments. The precursors and productions are extracted as ion chromatograms and evaluated using a coelution score. The software calculates a number that accounts for abundance, peak shape (symmetry), peak width, and retention time. The resulting compounds were reviewed in the Mass Hunter Qualitative version, and unqualified features were manually removed. Mass Hunter Qualitative results and qualified features were exported as a.cef file.

### Bioinformatic analyses

**Fig. 2:**
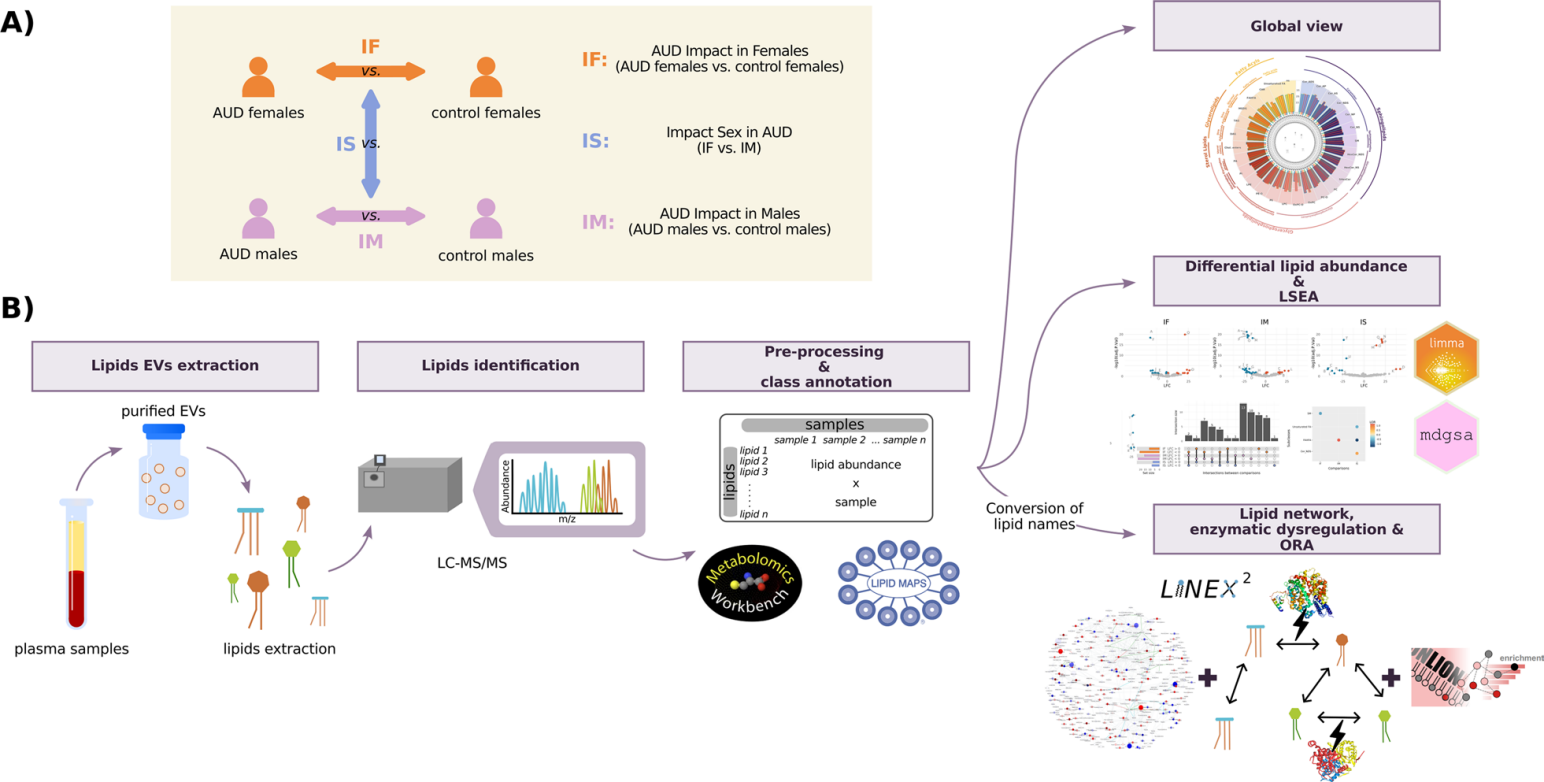
Experimental design and lipidomic workflow. **A** Description of the experimental groups and the comparisons made. **B** In the lipidomic workflow, lipids were extracted for quantification and identification through LC–MS/MS after isolating EVs from human plasma. Following data normalization and lipid class annotation, exploratory and differential analyses assessed lipid abundance. A class enrichment analysis was also performed. The LINEX^2^ platform provided: (i) a reaction global network, (ii) the subgraph with the most significant average substrate-product change, and (iii) a target lipids list for further enrichment analysis using LION-web

The strategy applied for this study was based on a transcriptomic analysis workflow. All bioinformatics and statistical analyses were performed using R software v.4.1.2 [25]. Figure 2A illustrates the experimental design, and Fig. 2B displays the whole lipidomic workflow.

### Data preprocessing

Data preprocessing included filter entities, normalization of abundance lipid matrix, and exploratory analyses. Mass Hunter Qualitative results (.cef file) were imported into Mass Profiler Professional (Agilent Technologies) for statistical analysis. Entities were filtered based on frequency, selecting those consistently present in all replicates of at least one experimental group. A percentile shift normalization algorithm (75%) was used, and datasets were baselined to the median of all samples. Normalized data were labeled according to negative and positive ion modes, and all data were consolidated into a single data frame. This step was followed by exploratory analysis using hierarchical clustering, principal component analysis (PCA), and box and whisker plots by samples and lipids to detect abundance patterns between samples and lipids and batch effects anomalous behavior in the data. At this point, anomaly-behaving samples and outliers (values that lie over 1.5 × interquartile range (IQR) below the first quartile (Q1) or above the third quartile (Q3) in the dataset) were excluded for presenting a robust batch effect with a critical impact on differential abundance analysis.

### Differential lipid abundance

The limma R package compared lipid abundance levels between groups [26]. P-values were adjusted using the Benjamini & Hochberg (BH) procedure [27], and significant lipids were considered when the BH-adjusted p-value ≤ 0.05.

### Class enrichment analysis

Class annotation was conducted using the *RefMet* database [28] and compared with the *LIPID MAPS* database [29]. Additional file 1: Table S2 details the description of abbreviations. Annotation was followed by ordering lipids according to the p-value and sign of the statistic obtained in the differential lipid abundance. Similar to a Gene Set Enrichment Analysis (GSEA) method, a class enrichment analysis was carried out using Lipid Set Enrichment Analysis (LSEA) implemented in the mdgsa R package [30]. The p-values were corrected for BH, and classes with a BH-adjusted p-value ≤ 0.05 were considered significant.

### Lipid network

The Lipid Network Explorer platform (LINEX^2^, https://exbio.wzw.tum.de/linex/) was used for lipid metabolic network analysis to gain insights into the sex-specific dysregulation of lipid metabolism in AUD patients [11]. For this purpose, single lipid species were considered as the sum or molecular species regardless of their retention time and ion mode acquisition. Therefore, before conducting the analysis, the lipid nomenclature was checked to ensure that most lipids in the study were included. This review was carried out using the MetaboAnalyst 5.0 platform [31] and the LipidLynxX Converter tool (http://www.lipidmaps.org/lipidlynxx/converter/) [32]. Additionally, a manual lipid-by-lipid revision was performed to ensure accuracy. LINEX^2^ analysis provided several results. The global lipid species network provides qualitative and quantitative associations between species based on defined reaction types and Spearman's correlation, respectively. In addition, changes in lipid levels between different experimental conditions can be derived from different statistical metrics. The subgraph with the most significant average substrate-product changes was obtained through a lipid network enrichment algorithm, which considered enzymatic multispecificity and generated hypotheses regarding enzymatic dysregulation. This algorithm consists of a local search approach that examines a search space greedily by iteratively testing local candidate solutions for the one with an optimal objective function. Candidate solutions are generated by applying one of three operations: node insertion, deletion, and substitution to the solution from the last iteration or a randomly selected subgraph in the first iteration. Lastly, LINEX^2^ provided a target lipids list derived from the lipids subgraph, which was utilized for an enrichment analysis using LION-web (http://www.lipidontology.com/) [33]. This enabled a more in-depth examination of the functional significance and potential biological implications of the identified lipid alterations.

### Comparisons

Three comparisons were performed to analyze differential lipid abundance (Fig. 2A):

i. AUD Impact in Females (IF) compares AUD females and control females (AUD.Females - Control.Females).
ii. AUD Impact in Males (IM) compares AUD males and control males (AUD.Males - Control.Males).
iii. Impact of Sex in AUD (IS) compares IF and IM (AUD.Females - Control.Females) - (AUD.Males - Control.Males).

Class enrichment analysis was assessed using the same three principal comparisons. LINEX^2^ analysis related to the global network was conducted using IF and IM comparisons, and the subgraph with the most significant average substrate-product changes was obtained using the control groups as a reference. The IF and IM comparisons were performed to identify the lipids whose abundance was affected by alcohol consumption separately in each sex. The IS comparison allowed us to identify the lipids whose abundance differed due to sex in the context of AUD.

The statistics used to measure the differential patterns were the logarithm of fold change (LFC) to quantify the effect of differential lipid abundance and the logarithm of odds ratio (LOR) to measure the enrichment of each functional class. A positive statistical sign indicates a higher mean for the variable in the first element of the comparison, whereas a negative statistical sign indicates a higher mean value for the second element. The IS comparisons focus on finding differences between female and male comparisons. Thus, a positive statistic may indicate either upregulation in females and downregulation in males or a higher increase or a lower decrease of the variable in AUD females. On the other hand, a negative statistic may indicate either upregulation in males and downregulation in females or a higher increase or a lower decrease of the variable in AUD males. In this comparison, the behavior of each lipid across the groups must be assessed a posteriori, examining female (IF) and male (IM) comparisons (Additional file 1: Figure S2).

### Web platform

All data and results generated in the different steps of bioinformatics strategy analysis are available on a web platform (https://bioinfo.cipf.es/sal-chronics), which is freely accessible to any user and allows the confirmation of the results described in this manuscript. The front end was developed using the Angular Framework, the interactive graphics used in this web resource have been implemented with plotly [34], and the exploratory analysis cluster plot was generated with the ggplot2 R package [35].

This easy-to-use resource is divided into seven sections: (1) a summary of analysis results; the detailed results of the (2) class annotation results; (3) exploratory analysis; (4) differential abundance between experimental groups; (5) LSEA results; and (6) metabolic lipid network results, where the user can interact with the web platform through graphics and tables and search for specific information related to lipid species or classes; and (7–8), which include methods, bioinformatics scripts, and supplementary material.

## Results

### Sex-based differences in lipid species and class lipid profiling of plasma EVs isolated from individuals with AUD

**Fig. 3:**
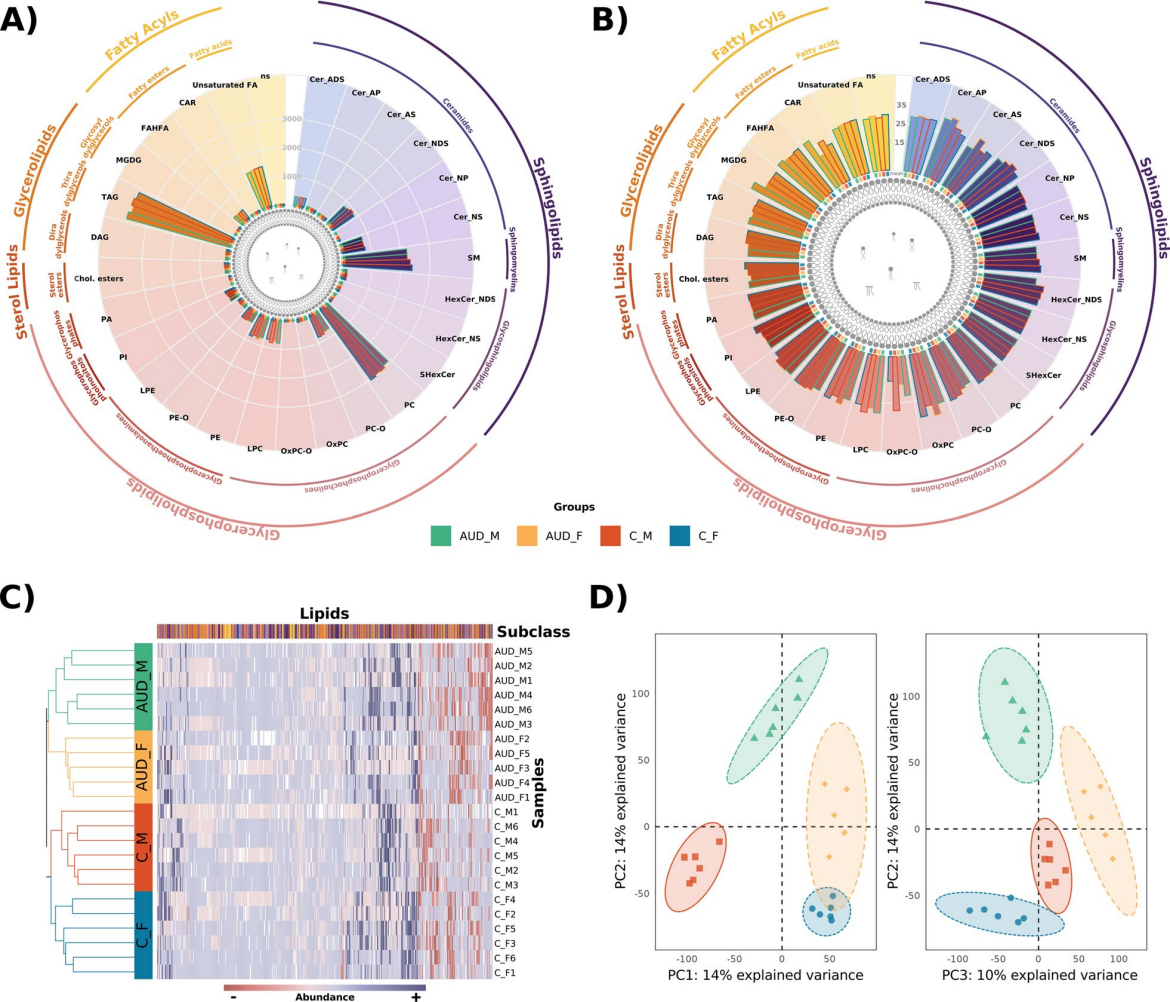
Lipid composition and distribution of EVs lipids in patient groups. Sum of the total (**A**) and median (**B**) of lipid abundance by lipid subclass across patient groups. Lipids were quantified as log2 of the identified peak area by LC–MS/MS analysis. According to the *RefMet* classification, the inner and outer lines of the radar plots indicate the lipid main class and super class, respectively. The color of the border of the bars indicates the patient group (AUD_M: green, AUD_F: yellow, C_M: orange, and C_F: blue). **C** Heatmap demonstrating the abundance patterns between lipids (columns) and samples (rows). Lipid subclasses and patient groups are indicated by the same colors previously assigned to them in the radar plots. Abundance levels are represented on a red-blue scale, where red indicates lower abundance and blue indicates higher abundance. **D** Principal component analysis (PCA) score plot showing 4 separate clusters according to the patient groups, which are indicated by the same color code as previously

The lipidomic profiles of plasma EVs from AUD and control females and males revealed 311 and 264 lipid compounds using negative and positive ion modes, respectively. After normalizing sample data, we labeled lipid species by ion mode. We employed RefMet and LIPID MAPS databases to classify all lipids (575 species) into different subclasses and their upper levels (super and main classes) (Fig. 3A and Additional file 1: Table S3). The descriptive analysis of lipid composition revealed enrichment of TAG, PC and SM subclasses in plasma EVs (Fig. 3A). Regarding the lipid abundance distribution of lipid subclasses (Fig. 3B), all patient groups displayed similar abundance profiles except for OxPC-O and CAR; however, hierarchical clustering of EV lipid species, regardless of their subclass, revealed distinct lipid profiles for the four experimental groups (Fig. 3C and D). The samples were separated by disease (AUD vs. healthy control) and sex (female vs. male) (Fig. 3C); moreover, part of the variance could be explained by sex (PC1) and disease (PC2) (Fig. 3D).

**Fig. 4:**
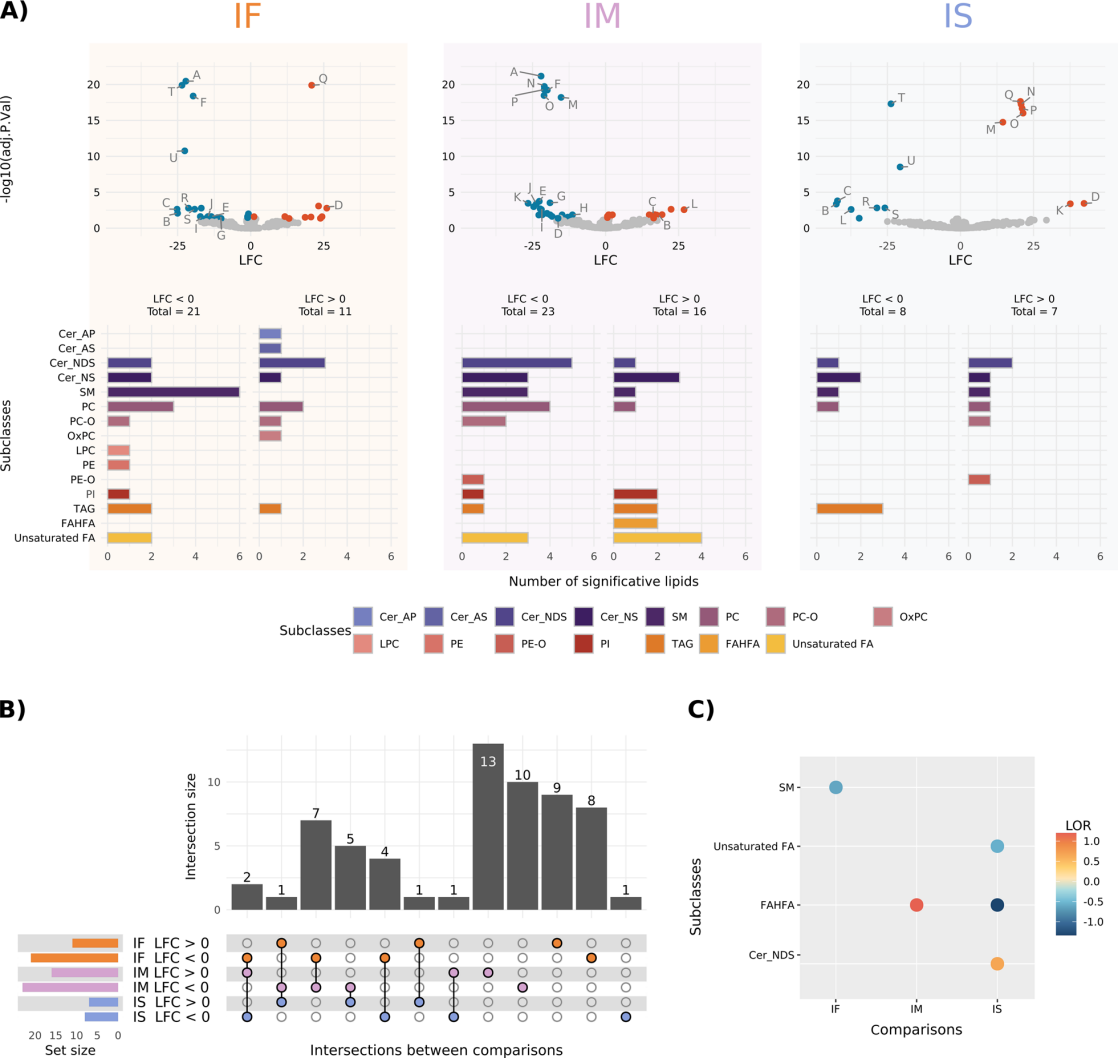
Summary of differential abundance analysis and molecular lipid profiles for each comparison. **A** Volcano plots summarize lipid data for IF, IM, and IS comparisons, while associated bar plots display significantly altered lipids classified by subclass and LFC. Significantly altered lipids with greater and lower abundance are shown in red and blue, respectively (p-value adjusted ≤ 0.05), in the volcano plots. Non-significant altered lipids are shown in gray. The capital letters in the volcano plots are the significantly altered lipids in at least two comparisons include, A: Cer_NDS d39:1_neg, B: Cer_NDS d42:2 RT:12.673_neg, C: Cer_NS d18:1_22:0_neg, D: Cer_NS d18:1_24:1_neg, E: Cer_NS d18:2_23:0_neg, F: EtherPC 16:0e_18:2_neg, G: FA 22:0 RT:6.523_neg, H: PC 32:3 RT:6.415_pos, I: PI 18:0_18:2_neg, J: SM d18:2_24:0_neg, K: Cer_NDS d42:1_neg, L: Cer_NS d18:1_24:0_neg, M: EtherPC 38:5e_neg, N: EtherPE 16:1e_22:6_neg, O: PC 18:2_20:4_neg, P: SM d37:2_pos, Q: Cer_NDS d18:0_18:0 RT:12.135_neg, R: PC 39:4_pos, S: SM d42:4_neg, T: TG 18:1_18:1_20:1_pos, U: TG 54:7_pos. Neg: negative ion mode, pos: positive ion mode. **B** Upset plot of the differential abundance analysis of lipids. Data from each comparison are separated according to the LFC sign. Horizontal bars indicate the number of significant lipids in each comparison (a specific color for each comparison). Vertical bars indicate the lipids included in the intersection of the groups denoted with a colored dot underneath. A colored dot under a bar indicates the specificity of the lipids in this group. **C** Analysis of the enriched significantly altered lipid subclasses by LSEA. Dot colors represent the sign and magnitude of the change (LOR). *IF* impact of AUD in females (orange), *IM* impact of AUD in males (purple), *IS* impact of sex in AUD (blue)

To assess significant variations in lipid abundance in plasma EVs, we carried out three comparisons: (1) AUD females vs. control females (IF), (2) AUD males vs. control males (IM), and (3) IF vs. IM (named “IS” to note the impact of sex). Figure 4A demonstrates that 32 and 39 lipid species displayed significant alterations (p-value ≤ 0.05) when comparing AUD females and males to controls, respectively. The IS comparison revealed fifteen significantly altered lipids, indicating a sex-specific response to AUD. Delving into the lipid subclasses noted in Fig. 4A to which significant lipids belong, we observed differences between all comparisons. Specifically, the Cer_AP and Cer_AS subclasses revealed significantly altered lipids with greater abundance in AUD females, while LPC and PE subclasses displayed significantly altered lipids with lower abundance in AUD females. The FAHFA subclass exhibited significantly altered lipids with greater abundance only in AUD males, whereas the PE-O subclass displayed one significantly altered lipid with lower abundance in AUD males. Notably, lipid species belonging to the PE-O subclass (Fig. 4A; label N) displayed lower abundance in AUD males and appeared significant with LFC > 0 in the IS comparison.

The analysis of significantly altered lipids shared between the different comparisons identified sex-specific lipid species (Fig. 4B). Specifically, we identified twenty-two female-specific lipid species, twenty-nine male-specific lipid species, and ten lipid species shared between the IF and IM comparisons. Considering these last ten lipid species, seven showed lower abundance in AUD females and males (Additional file 1: Table S4). The remaining three lipid species displayed the opposite abundance and significant alterations in the IS comparison (Additional file 1: Table S4). Furthermore, 15 lipids exhibited sex-based differences (IS comparison) in the AUD patients; some displayed significant alterations in the IF and/or IM comparisons, while one lipid displayed significant alterations in the IS comparison.

The LSEA results displayed a significant enrichment of the SM subclass in the IF comparison, with a lower representation in AUD females than control females (negative LOR value) (Fig. 4C). The IM comparison in the LSEA results suggested that the FAHFA subclass had greater representation in AUD males than control males (positive LOR value). We also observed a significantly higher enrichment of the Cer_NDS subclass in AUD females compared to AUD males; however, we also observed a significantly higher enrichment of Unsaturated FA and FAHFA subclasses in AUD males compared to AUD females (SI comparison).

### Sex-based differences in the lipid network of plasma EVs isolated from AUD patients

**Fig. 5:**
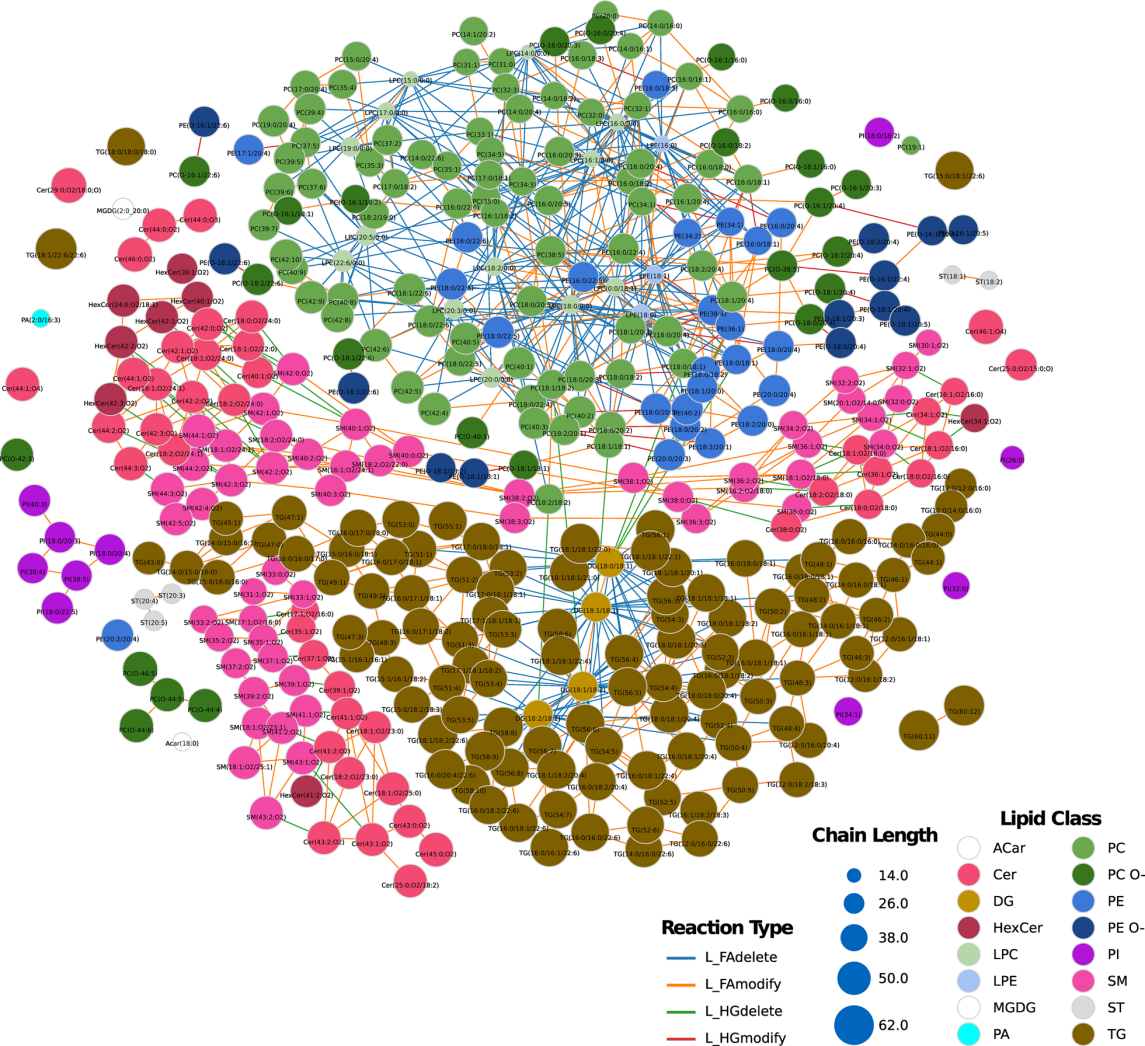
LINEX^2^ lipid network based on LC–MS/MS data. Colored spherical nodes depict lipid classes. Edge colors indicate the type of reaction connecting nodes. For further exploration and analysis, an interactive version of the network, along with all other LINEX^2^ analyses, are accessible in the web-platform http://bioinfo.cipf.es/sal-chronics/lipid_net.html

**Fig. 6:**
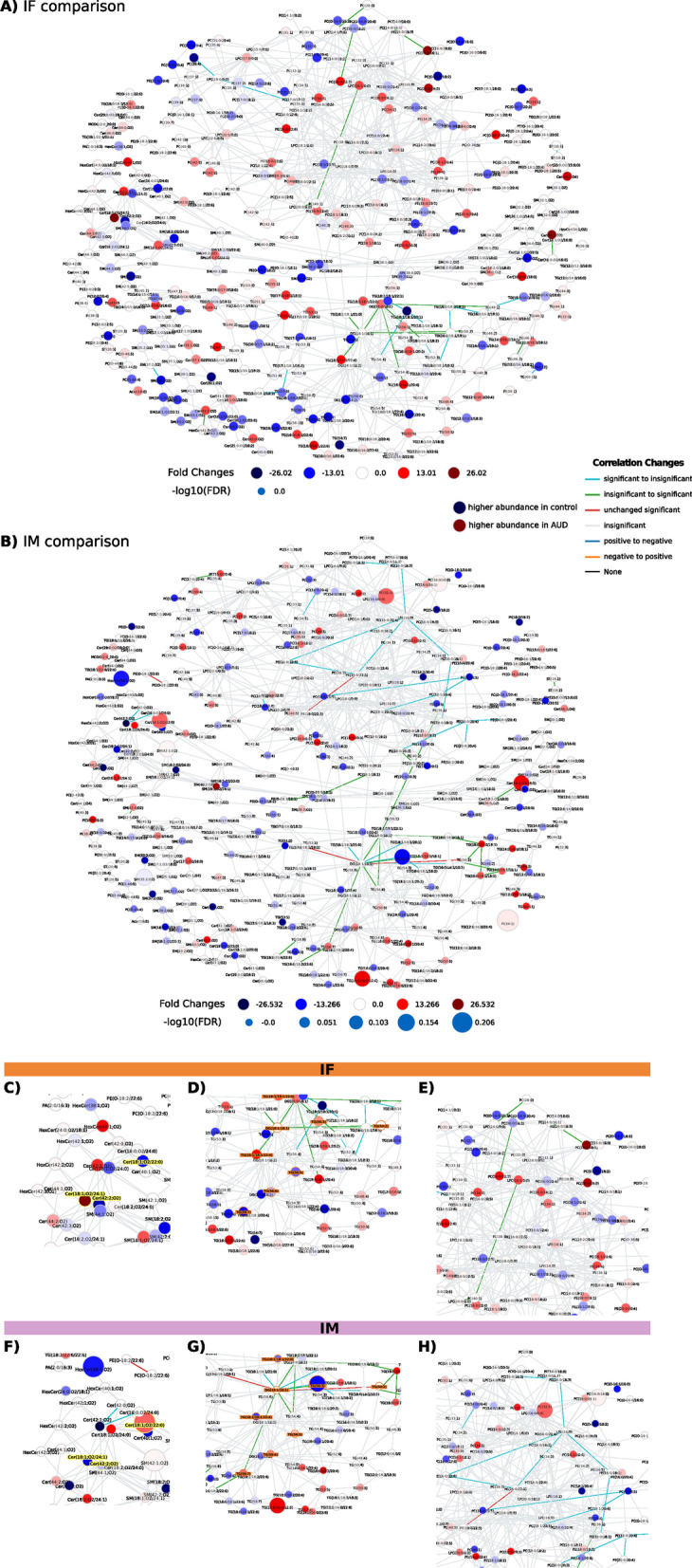
Lipidomics data visualized with LINEX^2^. Global lipid network visualization for IF (**A**) and IM (**B**) comparisons. **C**–**H** Magnified network views of specific lipids for IF (**C**–**E**) and IM (**F**–**H**). *IF* impact of AUD in females, *IM* impact of AUD in males. Red spherical nodes represent lipids with a positive LFC (higher abundance in AUD), whereas blue nodes indicate a negative LFC (higher abundance in control). The spherical node sizes indicate the −log10 FDR corrected p-values of lipid species between AUD and control females and males (a larger node size represents a higher level of statistical significance). Edges are colored by correlation changes for lipids from AUD patients to healthy individuals: negative to positive (significant correlation in both groups, < 0 in AUD and > 0 in control), positive to negative (significant correlation in both groups, > 0 in AUD and < 0 in control), significant to insignificant (significant correlation in AUD, insignificant in control), unchanged significant (significant in both groups, either both > 0 or both < 0), insignificant (uncorrelated in both groups), and insignificant to significant (insignificant in AUD, significant in control). Lipid network and other LINEX^2^ analyses can be explored in an interactive version, available as in the web-platform http://bioinfo.cipf.es/sal-chronics/lipid_net.html

LINEX^2^ aims to obtain a biological interpretation from lipidomics data. Figure 5 represents the global network of lipid species, which provides qualitative associations between species based on defined reaction types. Most reactions relate to fatty acid modification/removal (orange and blue edges). Figure 6 depicts similar qualitative associations as Fig. 5 while also providing quantitative information regarding alterations in lipid levels between AUD and control females (IF) (Fig. 6A) and males (IM) (Fig. 6B). The colored spherical nodes represent higher lipid abundance; therefore, the IF network (Fig. 6A) reveals lipids with increased abundance (not significant) in control females (blue nodes) and AUD females (red nodes) with a uniform distribution within the network; however, we also observed abundant lipids in control males (Fig. 6B, blue nodes). Analysis of the abundance lipids in AUD males (larger spherical nodes) also indicates statistical significance. The edge color in the network indicates the correlation change of the reaction connecting two nodes. Figure 6A, B reveals distinct patterns between the sexes, with some lipids exhibiting opposing LFC values. The magnified network view, represented by the lipid species Cer(18:1;O2/24:1), Cer(18:1;O2/22:0), and Cer(42:2;O2) (Fig. 6C and F), denoted as Cer_NS d18:1_24:1_neg, Cer_NS d18:1_22:0_neg, and Cer_NDS d42:2 RT:12.673_neg (Fig. 4), also confirmed sex significant differences. The lipid DG(18:1/18:1) (highlighted node, Fig. 6D and G) showed significant connections with several other lipids, displaying different correlations in the IF and IM comparisons. For instance, the interaction of DG(18:1/18:1) with TG(18:1/18:1/21:0) revealed a non-significant correlation in AUD females but a significant correlation in control females in the IF comparison. In the IM comparison, this correlation is significant for AUD males but not for control males. Furthermore, the global network also showed notable differences in the correlations between both sexes (Fig. 6E and H). In females, green edges indicate significant reactions in control patients (not significant in AUD females); in contrast, males showed an opposite network zoomed view (blue edges).

### Sex-based differences in lipid enzymatic dysregulation of plasma EVs isolated from AUD patients

**Fig. 7:**
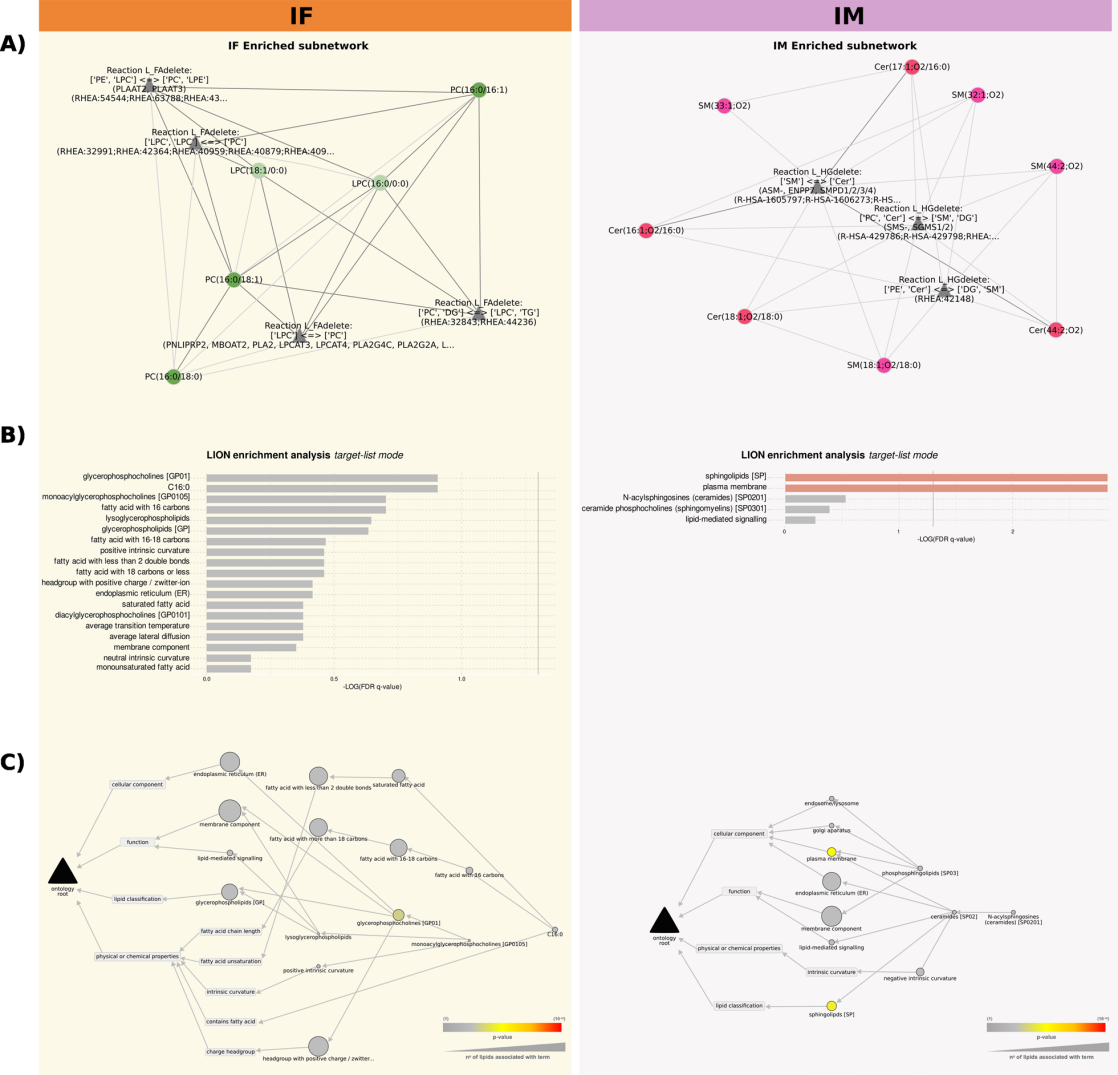
Enrichment subnetworks generated by LINEX^2^ based on global networks. **A** LINEX^2^ enrichment subnetworks for the IF and IM comparisons. IF—impact of AUD in females (orange); IM—impact of AUD in males (purple). Spherical nodes represent lipid species, and triangular nodes represent reaction type. **B** The most enriched ontology terms result from using the lipids in the subnetworks as targets in the target list mode. **C** A hierarchical network displaying the most enriched ontology terms results from using the lipids in the subnetworks as targets in the target list mode. The raw p-value scales the node colors, and the node size indicates the number of lipids involved in each ontology term

Using lipid class reactions from common metabolic databases through a network enrichment algorithm [11], we can determine enzymatic dysregulation from our EVs lipidomics data. Figure 7 depicts the enrichment networks generated by LINEX^2^ based on the global networks (Figs. 5 and 6). Figure 7A highlights the most dysregulated subnetworks between AUD and control females and males. The resulting subnetworks include only PC and LPC lipid species in female patients and Cer and SM in male patients, suggesting that enzymatic dysregulation participates in different biochemical reactions in the different sexes, transforming the lipid species into each other.

We identified differences between the sexes in LION enrichment analysis, using the lipids in the subnetwork as targets in target list mode (Fig. 7B). Female patients exhibited ontology terms related to membrane activity and stability, such as “positive intrinsic curvature”, “headgroup with positive charge/zwitter-ion”, “lipid-mediated signaling”, and “endoplasmic reticulum”. These concepts normally associate with the lipid class term “glycerophosphocholine”; the terms related to this class became enriched. The two most significant terms in male patients were “sphingolipids” and “plasma membrane”, which related to cell membrane and lipid signaling pathways. Female patients exhibited ontology terms related to the “positive intrinsic curvature” of the membrane, while male patients presented “negative intrinsic curvature” terms (Fig. 7C); interestingly, both terms relate to EV biogenesis. Thus, the properties of the lipidome assigned by the LION algorithm suggest alterations of lipids involved in membrane remodeling and lipid-mediated signaling in EVs from AUD patients, with a different pattern observed between the sexes.

### Web platform

The web platform (https://bioinfo.cipf.es/sal-chronics) contains detailed information regarding the complementary computational approaches involved in this study. This resource includes statistical indicators of each performed analysis, which users can explore to identify their profiles of interest. This open resource hopes to contribute to data sharing between researchers, elaborating innovative studies, and discovering new findings.

## Discussion

### Unveiling the lipid landscape in AUD

Preclinical studies have highlighted the importance of improving our understanding of the biological and metabolic pathways involved in AUD to promote the development of new therapeutic and diagnostic methods. While many related studies have focused on the use of EV-resident microRNAs and proteins as plasma biomarkers, our results demonstrated, for the first time, that LPC and PC lipids (and enzymes such as phospholipases and acyltransferases) suffer from changes associated with cancer progression and neuroinflammation in female AUD patients. Moreover, male AUD patients exhibit dysregulation of Cer and SM lipid species (which involve sphingomyelinases, sphingomyelin phosphodiesterase, and sphingomyelin synthase), which potentially contributes to ethanol-induced hepatotoxicity. Additionally, computational analyses highlight sex-specific variations in EV lipids that play roles in vesicle fusion processes.

Considering that most, if not all, cells in the human body secrete EVs into circulating bodily fluids, the characterization of EV lipid profiles could provide information regarding the cell/tissue of origin and their functional state [36]. The distribution of lipid species in absolute amounts highlighted PC, SM, and TAG as the most abundant lipid subclasses. Whereas PC represents an abundant lipid subclass in EVs derived from neural cells [37], the SM subclass participates in EV biogenesis and is among the most abundant classes in brain-derived EVs [38, 39]; moreover, we identify the novel lipid species SM d18:2_24:0 as a potential biomarker in female and male AUD patients. The presence of the TAG lipid subclass in EVs could arise from the secretory autophagy pathway [40]; in addition, TAG could become transferred from lipoproteins to exosomes once released into the bloodstream [41], suggesting the absence of lipoprotein contamination during the EV isolation procedure [9].

### AUD induced sex-based differences in lipid profiles related to EVs biogenesis and may underlie inflammatory and neurodegenerative responses

We previously reported that acute ethanol intoxication induced the enrichment of distinct plasma EV lipid species (e.g., LPC, PA, FAHFA) in human female adolescents compared to males; these lipid classes participate in the formation, release, and uptake of EVs and immune response activation [9]. Following the same sex-based differential analysis in AUD patients, our current results indicate a lower abundance of the LPC and PE subclasses in AUD females than in healthy individuals. LPC, which is enriched in EVs, is related to pro-inflammatory functions and participates in EV biogenesis [42]; moreover, LPC promotes demyelination by activating CNS inflammatory responses and inducing microglia pyroptosis [43]. Indeed, alcohol-induced pro-inflammatory molecules in the periphery may provoke neuroinflammation by crossing the brain-blood barrier [44]. A general decline in plasmalogen lipids (mainly PC and PE subclasses) has been described in multiple brain regions in Alzheimer's disease [37], which could associate with increased oxidative stress, inflammatory responses, and neuronal cell death [45, 46]; however, additional studies have reported high and low levels of PC and PE in highly metastatic breast cancer, respectively [47]. In addition, our results demonstrated that most ceramide lipid species (e.g., Cer_NS d18:1_24:1, Cer_NS d18:1_22:0, and Cer_NDS d42:2 RT:12.673) exhibited sex-specific abundances. The subclasses Cer_AP and Cer_AS displayed a greater abundance in AUD females, whereas some lipids belonging to Cer_NS and Cer_NDS displayed lower abundance. An increase in Cer_AS species along with a decrease in Cer_NS and Cer_NDS has been previously described in a mouse model of metachromatic leukodystrophy, suggesting that alpha-hydroxylation of ceramides may play a role in the brain pathology of this disease (e.g., demyelination and motor dysfunction) [48].

The Fatty Acids main class has been associated with inflammation [49] and neurotransmitter release [50] through cell surface and intracellular receptors, thereby being linked to the modification of membrane composition, cell signaling, gene expression, and lipid mediator production [49]. Our results revealed that unsaturated FA subclass (main class Fatty acids) had a negative LOR in the IS comparison, indicating class enrichment in AUD males compared with females. FAs have been implicated in neural cell pathology in lysosomal storage diseases, including metachromatic leukodystrophy, which is characterized by lipid accumulation in the brain, spinal cord, and peripheral nerves [48]. Furthermore, although the FAHFA subclass emerges as a significantly more abundant lipid in AUD males, we know little regarding the involvement of FAHFA in biological processes other than its anti-inflammatory role [51].

### Lipid network enrichment unveils intriguing sex-based variations in the pathology linked to AUD

Incorporating LINEX^2^ lipid network enrichment into our data provided the basis for a knowledge-driven integration of lipidomics with proteomics data by connecting enzymatic activity to lipid species [11]. The resulting network analysis revealed more significant substrate-product changes in AUD females for reactions involving the LPC and PC subclasses, including phospholipases and acyltransferases (e.g., LPCAT3/4). The upregulation of enzymes such as LPCAT1 has been reported in human colorectal adenocarcinoma [52] and metastatic prostate cancer [53], suggesting the involvement of the LPC metabolism in cancer progression. In addition, PLA2-activated neuroinflammatory pathways (through the upregulation of the oxidative stress status) become induced by binge alcohol drinking in adult rats and in organotypic hippocampal-entorhinal cortical slice cultures [54]. Our results also demonstrated that PLA2G2A becomes upregulated in AUD females; this enzyme, which possesses lysophospholipase, transacylase, and PLA2 activities [55], plays an antimicrobial role by degrading bacterial membranes and releasing pro-inflammatory eicosanoids from inflammatory cell EVs [56].

We also observed the enzymatic dysregulation of Cer and SM lipid species in AUD males. Previous studies reported alterations in the levels of various sphingolipids (including Cer and SM) in human chronic alcohol-related liver disease [57] and individuals with high alcohol consumption [58]. The enzymes involved in these substrate-product reactions—the sphingomyelinases (e.g., ASM, ENPP7, SMPD family, and SGMS1)—have been linked to chronic alcohol consumption [59]. In addition, recent studies revealed increased sphingomyelinase activity in ethanol-treated microglial cells [60] and high sphingomyelinase protein levels associated with alcoholic liver disease [61]. Since the enzymes involved in sphingolipid metabolism may mediate ethanol's hepatotoxic effects [62], ASMase activation and C16-ceramide generation could sensitize hepatocytes to the effects of TNF-α [63]. In agreement with our results, a sex-based evaluation by Mühle et al. reported high levels of serum ASMase activity in alcohol-dependent male patients [64].

### Sex-based differences in the properties of lipids associated with membrane remodeling and lipid-mediated signaling in EVs from AUD patients

As lipids exhibit many structural and signaling functions, the biosynthesis of lipids and changes to biophysical properties must be considered. We performed a comprehensive computational lipidomic analysis using network-based and lipid property-related methods through the LION algorithm to evaluate membrane remodeling and lipid-mediated signaling in EVs. Interestingly, our results demonstrated LION-term enrichment featuring “positive intrinsic curvature” in AUD females but “negative intrinsic curvature” in AUD males. Lipids with positive intrinsic curvature (such as LPC) hinder stalk formation during vesicle fusion [65] to facilitate fusion pore expansion [66]. While lipids with greater negative curvature (such as PE and DAG) represent critical players in fusion, lipids of lesser negative curvature (such as phosphatidic acid) generally play modulatory roles [67]. Lipids with negative curvature (such as oleic acid or DAG) significantly influence vesicle fusion processes [67, 68] and tend to promote stalk formation and inhibit pore expansion [69]. Notably, the formation and expansion of fusion pores during SNARE-dependent vesicle fusion remain essential for neurotransmitter release and vesicle recycling during exocytosis [70].

### Sex-specific lipidomic profiles suggest distinct mechanisms of alcohol-induced brain injury with direct therapeutic implications

Dysregulation of glycerophospholipid and sphingolipid metabolism, the most altered lipid classes in our study, underlies the disproportionate atrophy of the brain white matter (WM) in patients with AUD. WM is largely composed of myelin, characterized by an increased representation of cholesterol, glycosphingolipids and sulfatides, as well as phospholipids [71]. Chronic alcohol consumption compromises the microstructural integrity of the WM through demyelination, dysmyelination and axonal degeneration, leading to widespread volume loss at the macrostructural level [72]. In fact, all studies with postmortem brain tissue from AUD patients or animal models conclude that the most affected regions showed a broad and significant decrease in glycerophospholipids and sulfatides, the most abundant and characteristic lipid subclass of myelin [73–75], as well as ceramides, polyunsaturated fatty acids and cholesteryl ester fatty acid chains [76].

Sexual dimorphism in WM atrophy has been widely described. Whereas some areas are more affected in males, others are more affected in females, with direct implications for the sex differences observed in behavioral patterns associated with alcoholism [77, 78]. This differential brain regional vulnerability may be due to differential expression of enzymes that mediate the biosynthesis and degradation of membrane phospholipids and sphingolipids in males and females across brain regions. In line with our results, transgenic mice overexpressing ASM have shown differential expression of ASM between males and females in different brain regions, effects that are associated with different emotional behavior; a depressive phenotype in males and a social anxiety disorder-like phenotype in females [79]. Indeed, in recent years, there has been evidence that ASM may play a role in the mechanism of comorbidity between AUDs and anxiety/depression [80]. For instance, during alcohol withdrawal, ASM levels gradually decrease in both sexes, but the positive correlation with withdrawal symptoms is stronger in males than in females [64]. These findings are consistent with reports from retrospective studies of coexistence patterns of AUD and depression in the development of depressive disorders. Females are more likely to experience depression preceding AUD, whereas males are more likely to develop depression resulting from AUD [81]. These sex differences have direct implications for the treatment of the comorbid depression in AUD. Interestingly, several antidepressants act as ASM inhibitors [82, 83]. However, the prescription of this type of drugs should be limited to those patients whose depressive trigger is characterized by a high ASM activity, and therefore, prove to be much more promising in the emotional behavior of males with AUD.

### Limitations and considerations

This study aimed to provide data regarding individual lipid species to support a rigorous lipidomic pathway analysis, as lipid species of the same class can behave differently, leading to distinct biological functions; however, this analysis does suffer from certain limitations. For instance, (i) a lack of standardization in lipid nomenclature and integration into computational tools (e.g., FAHFA displays significant abundance but may not be included in the LINEX^2^ software); (ii) lipid databases (e.g., LIPID MAPS and HMDB) contain general information regarding lipid class biology; and (iii) LINEX^2^ details lipid species and their enzymatic activity, although this software package does not allow complete control and provides aleatory results based on the algorithm. Of additional note, the EVs used in this study have sizes and protein marker expression profiles similar to exosomes; however, we cannot currently specifically identify them as exosomes.

### Perspectives and significance

Given the role of sex differences in modulating vulnerability in AUD, our findings underscore the presence of sex-based differences in EV lipidomic profiles induced by AUD. These distinctions, evident in lipid network analysis and enzymatic dysregulation, highlight the innovative nature of our study. It employs a comprehensive bioinformatic strategy to explore the sex-specific effects of ethanol on lipidomic profiles, providing new insights into lipid metabolism. These findings suggest that AUD exerts diverse influences on the lipidome of EVs based on sex, emphasizing the critical role of sex-specific biomarkers (e.g., PC 16:0_16:1 in females, PI 34:1 in males, see Additional file 1: Table S4). Notably, dysregulation of glycerophospholipid and sphingolipid metabolism revealed a tendency toward phospholipid-mediated neuroinflammation in females and sphingolipid-mediated neuroinflammation in males. This knowledge not only advances our understanding of the intricate interplay between AUD and lipid metabolism, but also offers novel perspectives that could guide personalized diagnostic and treatment strategies.

## Conclusions

In conclusion, this study employed an innovative strategy based on a network enrichment algorithm to gain insight into the sex-specific dysregulation of lipid enzymatic reactions in AUD patients. Our findings unveiled sex-based differences in lipid profiles related to EV biogenesis that may underlie inflammatory and neurodegenerative responses. These methodological advancements have deepened our understanding of lipid metabolism and the associated regulatory mechanisms, facilitating the identification of novel lipid targets and the potential discovery of sex-specific clinical biomarkers for AUD.

### Supplementary Information

**Additional file 1: Figure S1.** Whole Western blots of CD9, CD63, CD81, and calnexin are shown. **Figure S2.** Bar chart representing the possible causes of a (A-E) positive or (F-J) negative LFC/LOR in the IS comparison, depending on the IF or IM comparison. **Table S1.** Characteristics of study individuals displaying chronic alcohol consumption. **Table S2.** Abbreviation of the different subclasses. **Table S3.** Classification by levels of all lipids in samples. **Table S4.** Lipids with significant differential abundance, separated by LFC.

## Data Availability

The datasets generated and analyzed during the current study and programming scripts are available in the Zenodo repository, 10.5281/zenodo.8360144, and in a web platform: https://bioinfo.cipf.es/sal-chronics.

## Abbreviations

ASM: Acid sphingomyelinase
DSM-5: Diagnostic and statistical manual of mental disorders fifth edition
ENPP7: Ectonucleotide pyrophosphatase/phosphodiesterase family member 7
HMDB: Human Metabolome Database
LPCAT 1/3/4: Lysophosphatidylcholine acyltransferases 1/3/4
PLA2: Phospholipase A2
SMPD: Sphingomyelin phosphodiesterase
SGMS1: Sphingomyelin synthase 1
SNARE: Soluble *N*-ethylmaleimide-sensitive-factor attachment protein receptor
