# Supplementary material for: Novel insight into the lipid network of plasma extracellular vesicles reveal sex-based differences in the lipidomic profile of alcohol use disorder patients
Source: Biol Sex Differ. 2024 Jan 25;15:10. doi: 10.1186/s13293-024-00584-5 (PMC10809459; doi:10.1186/s13293-024-00584-5)
Supplement: Supplementary file 1 — Additional file 1: Figure S1. Whole Western blots of CD9, CD63, CD81, and calnexin are shown. Figure S2. Bar chart representing the possible causes of a (A-E) positive or (F-J) negative LFC/LOR in the IS comparison, depending on the IF or IM comparison. Table S1. Characteristics of study individuals displaying chronic alcohol consumption. Table S2. Abbreviation of the different subclasses. Table S3. Classification by levels of all lipids in samples. Table S4. Lipids with significant differential abundance, separated by LFC. [file 13293_2024_584_MOESM1_ESM.pdf]

Supplementary Material

Figure S1. Whole Western blots of CD9, CD63, CD81, and calnexin are shown.

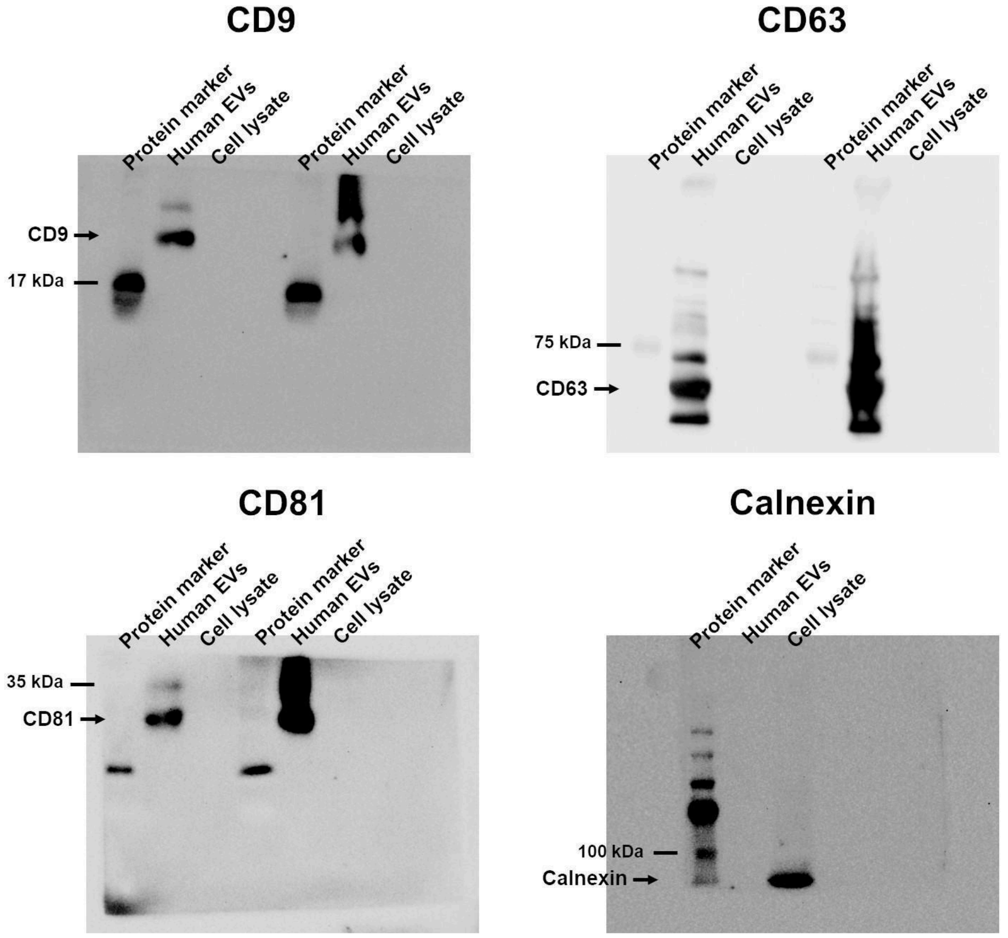

Figure S2. Bar chart representing the possible causes of a (A-E) positive or (F-J) negative LFC/LOR in the IS comparison, depending on the IF or IM comparison.

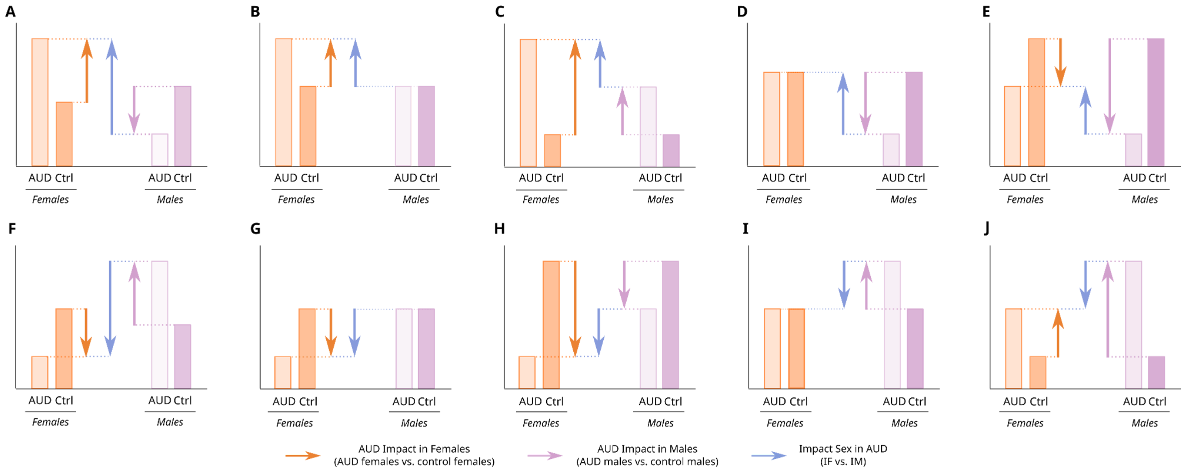

Table S1. Characteristics of study individuals displaying chronic alcohol consumption.

PARAMETER	Men (n=6)	Women (n=5)	*P*
**Age (years)**	47.83 (9.89)	40.00 (12.81)	0.28
**Total bilirubin (mg/dL)**	0.44 (0.17)	0.42 (0.21)	0.85
**AST (U/L)**	33.67 (25.28)	27.20 (9.31)	0.58
**ALT (U/L)**	27.17 (13.96)	34.20 (24.42)	0.72
**FA (U/L)**	94.83 (32.74)	64.60 (13.37)	0.09
**LDH (U/L)**	163.17 (22.39)	184.00 (56.45)	0.42
**GGT (U/L)**	138.83 (178.99)	70.00 (79.78)	0.45
**Proteins (g/dL)**	7.24 (0.37)	6.91 (0.81)	0.40
**Albumin (g/dL)**	6.44 (1.28)	5.21 (1.31)	0.15
**Ferritin (ng/mL)**	438.12 (389.52)	270.82 (422.91)	0.51
**Hemoglobin (g/dL)**	14.93 (1.17)	13.50 (1.24)	0.08
**Hematocrit (%)**	42.97 (3.13)	32.99 (16.75)	0.83
**MCV (fL)**	99.53 (10.76)	94.86 (5.87)	0.41
**MCH (pg)**	34.64 (4.02)	31.90 (2.78)	0.23
**Leukocytes (x10^3 cel/µL)**	7.70 (2.97)	7.42 (1.76)	0.86
**Neutrophils (x10^3 cel/µL)**	4.62 (1.87)	4.48 (1.58)	0.89
**Lymphocytes (x10^3 cel/µL)**	2.22 (0.95)	2.03 (0.49)	0.71
**Platelets (x10^3 cel/µL)**	243.33 (81.46)	247.80 (66.60)	0.92
**Total cholesterol (mg/dL)**	196.83 (28.46)	204.90 (36.22)	0.69
**Triglycerides (mg/dL)**	135.08 (114.40)	127.20 (35.45)	0.89
**Prothrombin activity (%)**	96.80 (4.15)	93.00 (4.83)	0.24
**APTT (segundos)**	35.08 (2.48)	35.95 (2.45)	0.61
**Fibrinogen (mg/dL)**	475.40 (115.58)	277.25 (55.05)	0.02
**D-dimer (µg/mL)**	0.36 (0.35)	0.54 (0.46)	0.54

Note: mean (standard deviation). AST: aspartate aminotransferase. ALT: alanine aminotransferase. AP: alkaline phosphatase. LDH: lactate dehydrogenase. GGT: gamma glutamyl transferase. MCV: mean corpuscular volume. MCH: mean corpuscular hemoglobin. APTT: activated partial thromboplastin time.

Table S2. Abbreviation of the different subclasses.

CAR	Acylcarnitine
Cer_ADS	Ceramide α-hydroxy fatty acid-dihydrosphingosine
Cer_AP	Ceramide α-hydroxy fatty acid-phytosphingosine
Cer_AS	Ceramide α-hydroxy fatty acid-sphingosine
Cer_NDS	Ceramide non-hydroxy fatty acid-dihydrosphingosine
Cer_NP	Ceramide non-hydroxy fatty acid-phytosphingosine
Cer_NS	Ceramide non-hydroxy fatty acid-sphingosine
Chol. esters	Cholesterol esters
DAG	Diacylglycerol
FA	Fatty acid
FAHFA	Fatty acid ester of hydroxyl fatty acid
HexCer_NDS	GlucosylCeramide/HexosylCeramidesnon-hydroxyfatty acid-dihydrosphingosine
HexCer_NS	GlucosylCeramide/HexosylCeramidesnon-hydroxyfatty acid-sphingosine
LPC	Lyso-phosphatidylcholine
LPE	Lyso-phosphatidylethanolamine
MGDG	Monogalactosyldiacylglycerol
OxPC	Oxidized phosphatidylcholine (OxPC)
OxPC-O	Oxidized etherphosphatidylcholine (OxEtherPC)
PA	Phosphatidic acid
PC	Phosphatidylcholine
PC-O	Etherphosphatidylcholine (EtherPC)
PE	Phosphatidylethanolamine
PE-O	Etherphosphatidylethanolamine (EtherPE)
PI	Phosphatidylinositol
SHexCer	SulfoglucosylCeramide/SulfohexosylCeramide
SM	Sphingomyelin
TAG	Triacylglycerol (TG)

Table S3. Classification by levels of all lipids in samples.

SUPER CLASS	N	MAIN CLASS	N	SUBCLASS	N
Sphingolipids	160	Sphingomyelins	79	SM	79
		Glycosphingolipids	10	HexCer_NDS	5
				HexCer_NS	3
				SHexCer	2
		Ceramides	71	Cer_ADS	11
				Cer_AP	3
				Cer_AS	3
				Cer_NDS	27
				Cer_NP	1
				Cer_NS	26
Fatty Acyls	64	Fatty acids	49	Unsaturated FA	49
		Fatty esters	15	CAR	2
				FAHFA	13
Glycerophospholipids	215	Glycerophosphates	1	PA	1
		Glycerophosphocholines	159	PC	102
				PC-O	27
				LPC	26
				OxPC	3
				OxPC-O	1
		Glycerophosphoethanolamines	44	PE	27
				PE-O	13
				LPE	4
		Glycerophosphoinositols	11	PI	11
Glycerolipids	130	Diradylglycerols	4	DAG	4
		Glycosyldylglycerols	1	MGDG	1
		Triradylglycerols	125	TAG	125
Sterol Lipids	5	Sterol esters	5	Chol. esters	5
ns	1	ns	1	ns	1
Total	575		575		575

Table S4. Lipids with significant differential abundance, separated by LFC.

Lipid	Ion mode	IF	IM	IS
Cer_AP t46:1	-	UP	NS	NS
Cer_AS d35:4	-	UP	NS	NS
Cer_NDS d18:0_18:0 RT:12.135	-	UP	NS	UP
Cer_NDS d18:0_18:0 RT:12.203	-	NS	DOWN	NS
Cer_NDS d34:1	-	UP	NS	NS
Cer_NDS d39:1	-	DOWN	DOWN	NS
Cer_NDS d40:1	-	NS	DOWN	NS
Cer_NDS d42:1	-	NS	DOWN	UP
Cer_NDS d42:2 RT:12.673	-	DOWN	UP	DOWN
Cer_NDS d42:2 RT:12.747	-	NS	DOWN	NS
Cer_NDS d46:1	-	UP	NS	NS
Cer_NS d18:1_18:0	-	NS	UP	NS
Cer_NS d18:1_22:0	-	DOWN	UP	DOWN
Cer_NS d18:1_24:0	-	NS	UP	DOWN
Cer_NS d18:1_24:1	-	UP	DOWN	UP
Cer_NS d18:2_23:0	-	DOWN	DOWN	NS
Cer_NS d42:3	-	NS	DOWN	NS
EtherPC 16:0e_18:2	-	DOWN	DOWN	NS
EtherPC 18:2e_20:4	-	UP	NS	NS
EtherPC 38:5e	-	NS	DOWN	UP
EtherPE 16:1e_22:6	-	NS	DOWN	UP
FA 17:0 RT:4.000	-	NS	DOWN	NS
FA 17:0 RT:4.252	-	NS	UP	NS
FA 18:2	-	NS	UP	NS
FA 19:0 RT:4.868	-	NS	DOWN	NS
FA 20:0 RT:4.411	-	NS	UP	NS
FA 20:0 RT:4.708	-	DOWN	NS	NS
FA 20:3	-	NS	UP	NS
FA 22:0 RT:6.523	-	DOWN	DOWN	NS
FAHFA 16:0_2:0	-	NS	UP	NS

Lipid	Ion mode	IF	IM	IS
FAHFA 18:0_6:0	-	NS	UP	NS
LPC 16:0/0:0	+	DOWN	NS	NS
OxPC 18:1_18:3+1O	-	UP	NS	NS
PC 16:0_16:0	-	NS	UP	NS
PC 16:0_16:1	-	UP	NS	NS
PC 16:0_18:0	-	UP	NS	NS
PC 16:0_22:6	+	NS	DOWN	NS
PC 17:0_20:4	-	DOWN	NS	NS
PC 18:2_20:4	-	NS	DOWN	UP
PC 19:2	+	NS	DOWN	NS
PC 32:3 RT:6.415	+	DOWN	DOWN	NS
PC 39:4	+	DOWN	NS	DOWN
PE 40:2	-	DOWN	NS	NS
PI 18:0_18:2	-	DOWN	DOWN	NS
PI 18:0_20:3	-	NS	UP	NS
PI 34:1	-	NS	UP	NS
SM d16:1_24:1	-	NS	UP	NS
SM d17:1_16:0	-	DOWN	NS	NS
SM d18:2_18:0	-	DOWN	NS	NS
SM d18:2_24:0	-	DOWN	DOWN	NS
SM d31:1	+	DOWN	NS	NS
SM d33:2	+	NS	DOWN	NS
SM d35:1 RT:9.090	+	DOWN	NS	NS
SM d37:2	+	NS	DOWN	UP
SM d42:4	-	DOWN	NS	DOWN
TG 16:0_18:1_20:4	+	NS	NS	DOWN
TG 18:1_18:1_20:1	+	DOWN	NS	DOWN
TG 46:2	+	NS	UP	NS
TG 48:4 RT:13.611	+	UP	NS	NS
TG 52:5 RT:14.049	+	NS	UP	NS
TG 53:5	+	NS	DOWN	NS

Lipid	Ion mode	IF	IM	IS
TG 54:7	+	DOWN	NS	DOWN
